# Memoir: template-based structure prediction for membrane proteins

**DOI:** 10.1093/nar/gkt331

**Published:** 2013-05-02

**Authors:** Jean-Paul Ebejer, Jamie R. Hill, Sebastian Kelm, Jiye Shi, Charlotte M. Deane

**Affiliations:** ^1^Department of Statistics, Oxford University, Oxford, OX1 3TG, UK and ^2^UCB Pharma, Slough, SL1 3WE, UK

## Abstract

Membrane proteins are estimated to be the targets of 50% of drugs that are currently in development, yet we have few membrane protein crystal structures. As a result, for a membrane protein of interest, the much-needed structural information usually comes from a homology model. Current homology modelling software is optimized for globular proteins, and ignores the constraints that the membrane is known to place on protein structure. Our Memoir server produces homology models using alignment and coordinate generation software that has been designed specifically for transmembrane proteins. Memoir is easy to use, with the only inputs being a structural template and the sequence that is to be modelled. We provide a video tutorial and a guide to assessing model quality. Supporting data aid manual refinement of the models. These data include a set of alternative conformations for each modelled loop, and a multiple sequence alignment that incorporates the query and template. Memoir works with both α-helical and β-barrel types of membrane proteins and is freely available at http://opig.stats.ox.ac.uk/webapps/memoir.

## INTRODUCTION

Membrane proteins mediate the exchange of signals and chemicals into every cell. Despite their pharmaceutical importance, few membrane protein crystal structures exist. The MPStruc database (http://blanco.biomol.uci.edu/mpstruc/) estimates that there are 383 unique protein structures in the protein data bank (PDB; as of 26 January 2013). The PDB itself contains ∼50 000 unique chains (1), meaning that despite comprising ∼25% of known sequences (2), membrane proteins constitute <1% of known structures.

In the absence of a crystal structure, the best source of structural information for a sequence is a homology model. A homology model is constructed by aligning the residues of the ‘target’ sequence onto the structure of a related ‘template’ protein. The accuracy of the model is determined by the quality of the alignment between the target and template, and by the coordinate generation method that turns this alignment into a 3D structure.

Owing to the small number of known membrane protein structures, a target membrane protein normally shares little sequence identity with any template, making accurate modelling challenging. Fortunately, structural constraints imposed on the protein by its biological membrane are thought to make membrane protein models more accurate than similarly remote globular protein models (3). The membrane also imposes constraints on sequence that can be used to improve the target–template alignment (4). Several web servers exist to produce homology models for globular proteins including HHpred (5), Swiss-Model (6) and RaptorX (7). However, no fully automated web server exists designed for general membrane proteins: at best this means that the constraints imposed by the membrane are not used in modelling, at worst the use of scoring functions designed for globular proteins may lead to distorted models.

**Figure 1.**
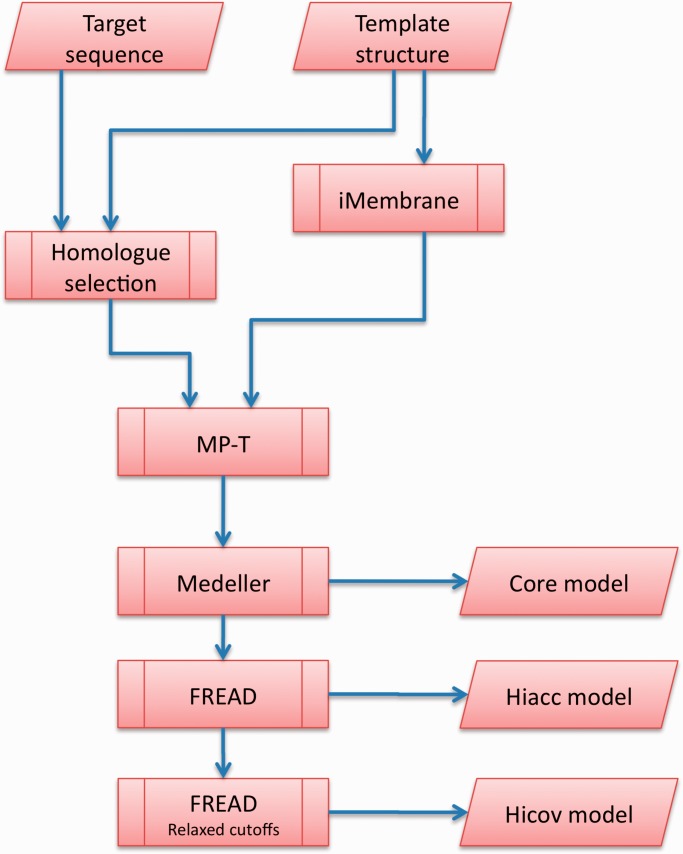
The Memoir pipeline. The user inputs are a target sequence to be modelled, and a template structure on which to base the model. The sequence of the template is annotated by iMembrane with structural information, such as position within the membrane and secondary structure. This annotation, together with a set of proteins that are homologous to the target and template, are aligned by MP-T. The alignment is used as a blueprint for model building by Medeller. The resulting ‘core’ model is available for download. Loops are then added to the core model to generate Memoir’s principal outputs: the high accuracy (Hiacc) and high coverage (Hicov) models.

Our Memoir web server is specifically designed for membrane proteins. An overview of Memoir’s pipeline is shown in Figure 1. First, the template protein is annotated with membrane-specific information by iMembrane (8). Next, homologous sequences are gathered for both the target and template proteins. These are aligned by MP-T (9), guided by the membrane information from iMembrane. Membrane information is again used in model building by the Medeller program (10), and the model is completed with a membrane protein-specific version of the FREAD loop-modelling method (11,12). These steps are described in more detail below.

## MATERIALS AND METHODS

### iMembrane: Annotating template membrane proteins

Template protein structures are annotated by the iMembrane program (8). iMembrane annotates each residue in the structure according to its accessible surface area, secondary structure, membrane positioning and extent of contact with lipids. iMembrane’s annotations are determined from molecular-dynamics simulations in the CGDB database (13). The use of molecular dynamics allows for distortions of the protein structure and membrane due to their mutual interaction. It also allows residues to be classified by the fraction of the simulation time for which they contact each part of a membrane lipid. Membrane lipids have hydrophilic heads and hydrophobic tails, so the local electrostatic environment of a residue is determined by the part of the lipid that it contacts.

### Homologue selection for alignment

The next step in the pipeline (Figure 1) is the collection of homologues of the target and the template using PSI-BLAST (14), running for five iterations on the Uniref90 database (15). A subset of the homologues is then selected as in (9). This selection procedure (see below) is a mixture of steps that filter out non-homologous sequences (such as a sequence identity cut-off), and steps that help the alignment algorithm (such as a cap on the maximum number of sequences).

Putative homologues are rejected if they have <15% sequence identity to the query, or if they are >3/2 or <2/3 the length of the query. The surviving homologues are made non-redundant at 80% sequence identity, and the homologues from the target and template are combined in equal numbers to prevent bias. This combined set is again made non-redundant. Up to 125 of the surviving sequences are randomly selected to help guide the target–template alignment.

### MP-T: Target–template alignment

The target and template are aligned with the MP-T sequence-structure alignment method. The MP-T algorithm first copies the annotation of the template on to each homologue. Subsequently every pair of sequences is aligned guided by these annotations. For example, a residue that is annotated as being in a transmembrane α-helix will rarely be aligned to a gap (indels are rare in transmembrane elements), and will be preferentially aligned to an amino acid type that is favoured in transmembrane helices.

The pairwise alignments are used to construct a guide tree to select homologues for a multiple alignment phase: only sequences judged by the guide tree to be descendants of the most recent common ancestor of the target and template are selected. Multiple alignment then proceeds using MP-T’s implementation of the T-Coffee objective criterion (16). This criterion attempts to make a multiple alignment that is as consistent as possible with the pairwise alignments.

### Medeller: Coordinate generation

The target–template alignment is then fed to Medeller for coordinate generation. Homology modelling is most effective in the middle of transmembrane sections, where membrane proteins are under the greatest structural constraints. The Medeller coordinate generation method builds models outwards from these constrained sections. Models consist of the protein backbone and C_β_ atoms, as well as the side chains of conserved residues. Model building stops when a local assessment of the quality of the sequence alignment suggests that structural similarity can no longer be assumed. This results in a ‘core model’, which is then extended by the FREAD fragment modelling method (Figure 1).

### FREAD: fragment modelling

FREAD searches a protein database for fragments of the appropriate length to fill gaps in a model. Potential matches are filtered based on the propensity for the un-modelled residues to assume the conformation required by the fragment. The remaining fragments are then ranked by how closely their termini match the flanking regions of the gap in the model.

Memoir generates two models, which differ in how highly scoring a database fragment must be before it is included in the model: one is termed the ‘high accuracy’ model (∼70% of the target sequence is modelled), the other the ‘high coverage’ model (∼76% of the target sequence). To produce the high-accuracy model, FREAD is run on a database of membrane protein fragments. The high coverage model includes additional lower scoring loops from the membrane fragment database as well as loops from a soluble fragment database. Both models include all major secondary structure elements.

### Web server usage

**Figure 2.**
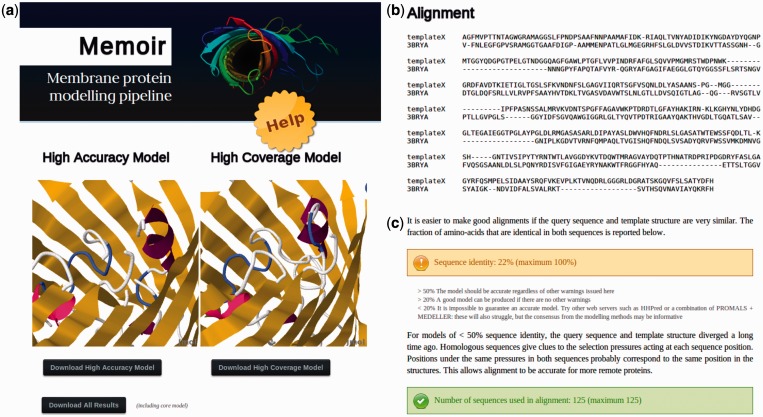
Parts of a Memoir results page: (**a**) two models are generated, one prioritizing accuracy (the ‘high accuracy’ model) and the other completeness (the ‘high coverage’ model). They are displayed in the Jmol 3d graphics viewer and are available for download in PDB format. Additional information on model creation can be downloaded using the ‘Download all results’ button. (**b**) Also displayed is the alignment between the target and template structure that was used in model building. (**c**) The alignment is accompanied by a guide to model quality, an extract of which is shown here. Values referenced in the guide, such as sequence identity, are calculated and displayed with traffic-light colour-coding (e.g. green for values that are likely to lead to a good model).

The Memoir server accepts a template structure in PDB format and a sequence to be modelled in FASTA format. The template can either be uploaded or specified by a PDB code. A typical query takes <1 h to run. An example results page is shown in Figure 2. Two models are produced: one with higher accuracy, and one with higher coverage. These are displayed in the Jmol 3D graphics viewer (17) and are available for download in PDB format (Figure 2a).

A proxy for the expected quality of a model is the quality of the corresponding target–template alignment. The results page displays this alignment (Figure 2b) together with a guide to model quality estimation based on alignment properties (an extract of which is shown in Figure 2c).

The generation of a homology model requires several programs, each of which produces its own output. A ‘Download all results’ button provides the supporting information for these methods. This information includes alternative loop structures for each loop modelled by FREAD, a Medeller model without fragment modelling (the ‘core’ model) and the full multiple sequence alignment from which the target–template alignment is inferred.

## RESULTS

The main source of error in homology models is inaccuracies in the target–template alignment (18). When tested against seven other methods on a set of 115 pairs of membrane proteins, MP-T produced alignments with the smallest fraction of misaligned residues (9). Reducing the fraction of misaligned residues allows better models to be built by coordinate-generation programs.

The most cited coordinate-generation software is Modeller (19). Medeller has been tested against Modeller on a data set of 616 target–template membrane protein pairs spanning a range of sequence identities (10). On average Medeller’s core models (i.e. the models before FREAD fragment modelling, see Figure 1) had a backbone root mean square deviation (RMSD) of 1.97 Å to the native structure, compared with 2.57 Å for Modeller. This trend was true at all levels of sequence identity and may be caused by distortions of the backbone introduced by Modeller’s probability density function, which is designed for soluble proteins.

When using different alignment methods with Medeller, it was found that models generated from MP-T alignments had marginally lower coverage, but significantly higher GDT_TS (20) than models from the next best alignment method (1/4 of models saw an increase in GDT_ TS of ≥4%) (9).

Memoir produces more complete models than those described above by augmenting the core. During this process the core is fixed, preserving the RMSD advantage that Medeller enjoys over Modeller. On a test set of 156 loops from 59 Medeller core models, loop modelling led to a high-coverage model that filled 150 of the loops. In 109 of 150 of these cases, the FREAD loop model was more accurate than Modeller’s *ab initio* loop model on the same set.

To illustrate Memoir’s use, models of the transmembrane domains of 15 membrane proteins were built using Memoir, HHpred and Swiss-Model’s automated mode (Table 1). Over the residues common to all three models Memoir had the lowest average RMSD (2.57 Å). In four cases, Memoir’s high accuracy model had <80% coverage, but the region that Memoir left un-modelled was modelled poorly by the other methods: seven of the eight fuller models built by HHpred and Swiss-Model had RMSDs of >5 Å.

**Table 1.** Comparison of models of 15 transmembrane domains built by Memoir (high-accuracy model), HHpred and Swiss-Model

Target/template	% id	% Cov^a^	RMSD^b^		
			Memoir	HHpred	Swiss-Model
2Q7MC/2H8AA	10	57	4.07	3.63	3.76
2JMMA/2LHFA	13	62	3.85	3.85	5.29
3GIAA/3L1LA	15	93	**3.97**	4.20	4.81
3O0RB/3MK7A	18	92	3.18	**2.64**	2.91
1OGVM/2AXTa	19	59	**2.60**	5.06	3.39
2VL0A/3RHWA	22	93	2.61	2.58	2.64
3BRYA/3DWOX	23	84	4.25	3.67	3.55
2WIEA/2X2VA	27	80	1.31	1.47	1.31
1YC9A/3PIKA	27	89	1.35	2.16	1.35
2D57A/2W2EA	31	97	**1.80**	2.06	2.02
2HYDA/3B60A	34	94	2.31	2.97	2.33
1L0LD/1ZRTD	35	89	1.30	1.54	1.28
1EZVE/2FYNC	47	65	**2.11**	3.72	3.01
1M56C/1OCCC	48	99	**1.10**	2.33	2.04
2QKSA/3SYOA	50	90	2.72	2.58	3.15
	Mean	83	2.57	2.96	2.86

An entry is in bold if the RMSD for the method is >0.2 Å lower than that of the next most accurate method.

^a^Coverage is assessed over the transmembrane domain.

^b^RMSD is assessed over common residues in all the models in the transmembrane domain.

## CONCLUSION

Memoir is currently the only web server designed for the homology modelling of general membrane proteins. Memoir works on all types of transmembrane protein (α-helical and β-barrel) and is easy to use. The main outputs of the server are two models in PDB format, one of which prioritizes model accuracy, and the other model completeness. Memoir’s results include supplementary information that could be used in manual model refinement, such as a multiple sequence alignment incorporating the target and template protein sequences and alternative conformations for each modelled loop. A video tutorial and a guide to the interpretation of results are provided.
